# Molecular Dynamics Simulations of Self-Assembling Colloids in Fed-State Human Intestinal Fluids and Their Solubilization of Lipophilic Drugs

**DOI:** 10.1021/acs.molpharmaceut.2c00710

**Published:** 2022-11-09

**Authors:** Albin Parrow, Per Larsson, Patrick Augustijns, Christel A. S. Bergström

**Affiliations:** † Department of Pharmacy, Uppsala Biomedical Center, Uppsala University, P.O. Box 580, SE-751 23 Uppsala, Sweden; ‡ The Swedish Drug Delivery Center, Department of Pharmacy, Uppsala Biomedical Centre, Uppsala University, P.O. Box 580, SE-751 23 Uppsala, Sweden; § Department of Pharmaceutical and Pharmacological Sciences, KU Leuven, O&N II Gasthuisberg, Herestraat 49, Box 921, 3000 Leuven, Belgium

**Keywords:** molecular dynamics simulations, human intestinal fluids, lipophilic drugs, micelles, lipophilicity

## Abstract

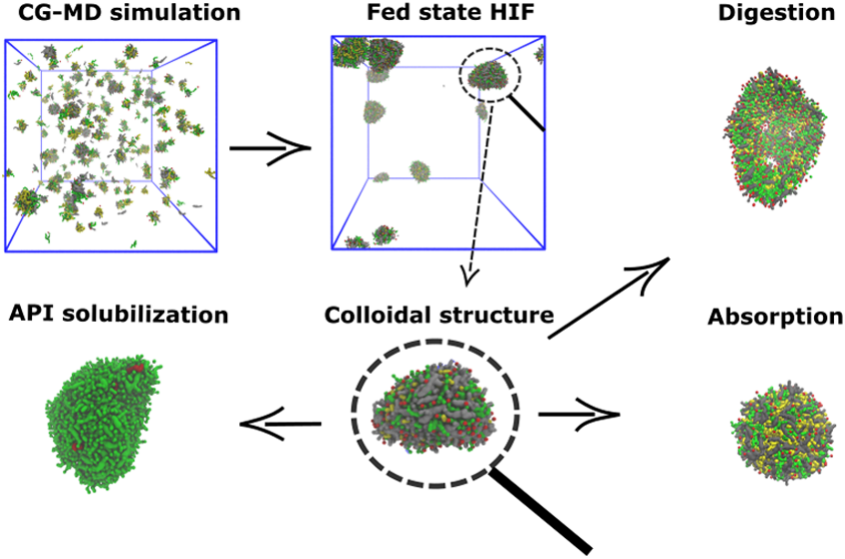

Bioavailability of oral drugs often depends on how soluble the active pharmaceutical ingredient is in the fluid present in the small intestine. For efficient drug discovery and development, computational tools are needed for estimating this drug solubility. In this paper, we examined human intestinal fluids collected in the fed state, with coarse-grained molecular dynamics simulations. The experimentally obtained concentrations in aspirated duodenal fluids from five healthy individuals were used in three simulation sets to evaluate the importance of the initial distribution of molecules and the presence of glycerides in the simulation box when simulating the colloidal environment of the human intestinal fluid. We observed self-assembly of colloidal structures of different types: prolate, elongated, and oblate micelles, and vesicles. Glycerides were important for the formation of vesicles, and their absence was shown to induce elongated micelles. We then simulated the impact of digestion and absorption on the different colloidal types. Finally, we looked at the solubilization of three model compounds of increasing lipophilicity (prednisolone, fenofibrate, and probucol) by calculating contact ratios of drug–colloid to drug–water. Our simulation results of colloidal interactions with APIs were in line with experimental solubilization data but showed a dissimilarity to solubility values when comparing fasted-/fed-state ratios between two of the APIs. This work shows that coarse-grained molecular dynamics simulation is a promising tool for investigation of the intestinal fluids, in terms of colloidal attributes and drug solubility.

## 1 Introduction

The most important administration route of small molecular drugs is oral. It is cost-efficient, making medicines available to a greater number of people, and gives the best patient compliance. However, over the last decades, the trend is toward more lipophilic drug candidates. In most cases, this lipophilicity goes hand in hand with a lack of solubility in water.^1−3^ For oral drugs, the active pharmaceutical ingredient (API) is absorbed into systemic circulation in the intestine. The solubility of the API in the intestinal environment is thus of great importance since only the free monomeric form of molecules is absorbed. After oral ingestion, the API needs to dissolve in the solvent, i.e., the intestinal fluid. The permeability of APIs through the small intestinal barrier can also be the rate-limiting step for drug absorption. This can also be influenced by the intestinal fluids, in a drug-specific manner.^4−6^ Human intestinal fluid (HIF) is mainly composed of water, hence the importance of API aqueous solubility.

However, other components in HIF make it more complex than plain water. Bile, which is made in the liver and stored in the gall bladder, is secreted into the duodenum. Bile is a fluid containing bilirubin, bile salts, phospholipids, and cholesterol, along with other components such as proteins and electrolytes.^7^ Bile salts are amphiphilic molecules, formed from a bile acid conjugated to an amino acid. In HIF, the most common type of bile salt is a cholate together with either taurine or glycine. The number of hydroxyl groups determines the type of cholate with the most common ones being taurocholate, taurochenodeoxycholate, taurodeoxycholate, glycocholate, glycochenodeoxycholate, and glycodeoxycholate.^8^

Depending on the ratio of bile salts to phospholipids,^9,10^ various colloidal structures form, ranging from mixed micelles to vesicles to droplets of micron size. These colloidal structures may also contain free fatty acids from the HIF. Food consumption of course plays a major role in the composition of the small intestine fluid content and consumed fats and glycerides affect the colloidal landscape. The consumption of food triggers more release of bile, thus elevating the concentration of both bile salts and phospholipids in the fed state.^11^ The concentrations of the multiple components in the HIF vary dynamically as digestion and absorption occur.

It is of great importance to determine the solubility of new APIs in HIF because of their role in drug absorption. In vitro testing is the most common approach, which uses media containing HIF components,^12^ such as fasted- and fed-state simulated intestinal fluid (FaSSIF and FeSSIF, respectively). However, computational techniques to predict solubility are useful because APIs are typically in limited supply and simulations reduce laboratory work. One such tool to estimate water solubility is quantitative structure–property relationships (QSPR), which uses molecular descriptors of the drug for prediciton.^13,14^ This approach can be used for screening compounds quickly and has reasonably accurate prediction.

Nevertheless, there is room for improvement and for better understanding of the results.^15^ To gain further insights into HIF and its solubilizing interaction with APIs molecular dynamics (MD) simulations have a potential usage. MD is a simulation technique based on Newton’s equation of motion, which calculates velocities of atoms over time depending on distances from other atoms, within a defined system.^16,17^ MD simulations can be very detailed, with several representations for different parts of an atom (first principles simulations),^18^ or with one representation for each atom (all-atom simulations).^19^ The drawback of detailed atomic simulation is the computational cost. For instance, an all-atom simulation duration is usually in the 100 ns range and the system size <20 nm box length, i.e., it results in a reduced time scale and system size. For longer simulations and for larger systems, coarse-grained (CG) MD can be used, in which several heavy atoms share a single representation.^20^ Different force fields can be more or less coarse-grained. For biological systems the Martini force field, with 3–4 heavy atoms per representation, is popular.^21^ MD simulations have been used to simulate intestinal fluid and bile components such as bile salts, phospholipids, and free fatty acids.^22−25^ A recent example is the study of Tunçer and Bayramoğlu, who have used a CG-MD model built upon the Martini force field to investigate the impact of free fatty acid type on colloidal structures in systems resembling fed-state HIF.^26^ Their study provides insights into micelle properties—such as molecular ordering and packing density—and how they relate to free fatty acid chain length and saturation.

In our previous work,^27^ we have simulated fasted-state HIF with concentrations of key components obtained from quantified, aspirated duodenal fluids from five healthy individuals (HVs),^8^ in an attempt to relate the simulation data of APIs and colloids to the API solubility in fasted HIF. In the current study, we used several sets of CG-MD simulations to describe the colloidal environment in fed-state HIF from the same HVs. We then used self-assembled colloids from our fed-state simulations, together with three different APIs and related these simulation results to the solubilizing capacity of the fluids. Finally, we investigated the impact of digestion and absorption on colloidal structures by additional time-resolved simulations.

## 2 Materials and Methods

### 2.1 Composition of HIF Used in Simulations

In a study by Riethorst et al.,^8^ fasted- and fed-state HIFs were collected from 20 HVs and the small molecular components of these fluids were quantified, specifically bile salts, free fatty acids, cholesterol, phospholipids, monoacylglycerol (MAG), diacylglycerol (DAG), and triacylglycerol (TAG). In our study, we used the concentration from five of these fed-state HVs to set up our simulations (Table 1). These concentrations were taken from pooled samples of different time points within 2 h after meal consumption. The five HVs were selected on the basis of their varying concentration of bile components in the fed state. The aim was to capture the interindividual variability in composition of fed HIF as much as possible.

**Table 1:** Concentrations (in mM) Used in the CG-MD Simulation, from Collected Fed-State HIF from Five HVs^8^

HV number:	3	6	9	16	20
bile salts	10.8	28.0	13.6	10.7	15.4
phospholipids	4.2	6.9	6.5	7.7	4.2
free fatty acids	11.1	13.8	44.9	38.4	27.2
cholesterol	0.5	1.0	0.3	1.7	0.5
monoacylglycerides	7.1	5.6	10.4	9.8	10.6
diacylglycerides	1.8	1.7	2.7	1.9	4.1
triacylglycerides	1.1	0.5	1.3	1.5	2.2

### 2.2 Topologies and Simulations Parameters

**Figure 1:**
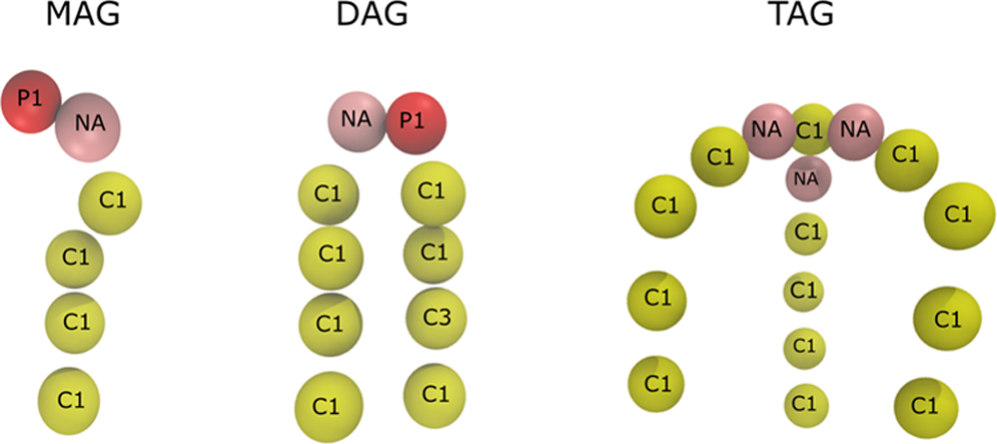
CG representations of mono-, di-, and tri-acylglycerides (MAG, DAG, and TAG, respectively); beads are labeled with bead types within the Martini force field.

The Gromacs software^28^ was used together with the Martini force field version 2.2^29^ for all simulations. All HV systems had cubic boxes of 45 nm side lengths. Taurocholate (TC), taurodeoxycholate (TDC), glycocholate (GC), and glycodeoxycholate (GDC) were used to represent bile salts with topologies taken from previous simulations of HIF and simulated intestinal fluids.^27,30^ Topologies for the remaining molecules (MAG, DAG, and TAG) were taken from the Martini website cgmartini.nl. Phospholipids were represented by single-tail lysophosphatidylcholine (PPC) and double-tail 1-palmitoyl-2-oleoyl (POPC) molecules. Fatty acids (FAs) were simulated with *n* = 16–18 carbons (palmitic – oleic acid). MAGs, DAGs, and TAGs hydrophobic tails were represented with four Martini beads, also corresponding to a chain length of 16–18 carbons (Figure 1). Standard, nonpolarizable Martini water beads were used as solvent. If freezing occurred in the systems, 10% of water beads were substituted with specific antifreeze beads. All simulations used simulation parameters recommended for the Martini force field with a leap-frog integrator for integration of the equations of motion. However, the antifreeze beads alone were not enough to prevent artificial nucleation from occurring within 2 μs in the system simulating the duodenal fluid of HV6. Due to the frequent freezing, a leap-frog stochastic dynamics (SD) integrator was used for this simulation instead. Simulations were performed either on supercomputers at the Center for High Performance Computing at the Royal Institute of Technology in Stockholm or on local GPU-servers.

### 2.3 Simulations of Fed-State HIF from Five HVs

To simulate the fed states of the five HVs, we performed three different simulation sets, using the same general simulation parameters in each. The idea behind this setup was to evaluate if the starting configuration impacts which colloidal structures are formed. Energy minimization and equilibration were performed as in our previous research,^27^ and the production run was set to 3 μs. The three different starting points are described in detail below.

Set 1 used a randomized initial distribution of all molecules in the simulation boxes. This is the same approach used in our previous research,^27^ and which is the most common starting point in MD simulations.

Set 2 used the same approach as set 1, but without glycerides. This was explored due to experimentally obtained data in the literature that reports the presence of fat droplets in fed-state HIF.^31^ These droplets are in the μm range, which is not suitable for our simulations. In set 2, we therefore made the assumption that the glycerides would stay in these droplets, with the result that the simulations focused on the smaller colloidal fraction.

Set 3 started from where the earlier fasted-state simulations ended. This is the natural state of the intestinal fluid when food is ingested. These simulations started with random distribution of the fed-state concentration molecules added to the last frame from the fasted-state simulations.

### 2.4 Simulations of Potential Impact from Digestion and Absorption

To understand how digestion impacts the colloidal structures formed, we isolated one colloid of each type with glyceride content (prolate micelles and vesicles) and exposed it to “manual” digestion. This was done by replacing all TAGs with one DAG and one free fatty acid. Since the number of beads in one TAG is equal to the number of beads in one DAG combined with one free fatty acid in our simulations, this was a relatively simple procedure. After these substitutions, the colloid was simulated in a water box (box length 20 nm) for 1500 μs. Afterward, we further digested the colloid by replacing all DAGs with two free fatty acids. Optimally we wanted to replace them with one MAG and one free fatty acid, but since the bead number does not add up for our topologies, we chose to go with complete digestion in the final step by releasing two free fatty acids. Following this second digestion step, the system was simulated for another 1500 μs.

To simulate absorption we followed a similar manual approach in which we removed free fatty acids from the simulations. To make sure that the system did not experience any large pressure fluctuations, we only removed five free fatty acids at a time, and simulated 2000 steps between each removal. In this way, 60% of the free fatty acids were removed, corresponding roughly to the difference seen in HVs when comparing intra-individually the highest to the lowest free fatty acid concentration at the time points in the fed state.

### 2.5 Simulations of API with Colloids

Next, we looked at the interactions of the APIs with the colloids that were assembled in the simulations. We chose one micelle and one vesicle to look at the role of the colloidal structures in the solubilizing capacity of the fluid. Colloids were isolated in new simulation boxes (box length = 20 nm) with added water beads, ions to neutralize the systems, and APIs. These newly formed systems were then simulated for a duration of 3 μs. During early equilibration, absolute position restraints were assigned to molecules within the colloid to inhibit the influence of pressure instabilities in the system. We worked with the same APIs as those studied in the fasted state: prednisolone, fenofibrate, and probucol.^27^ The prednisolone topology was taken from Estrada-López et al.^32^ who parameterized and used it for simulations to observe prednisolone interactions with phospholipid membranes. Fenofibrate and probucol were parameterized and used in our previous research, in which we also reduced the Lennard-Jones potential to inhibit excessive, unphysical self-aggregation.^27^ The APIs were added in four different concentrations (*n* = 2, 5, 10, and 50). The concentration levels of *n* = 2, 5, and 10 roughly represent physiologically relevant ones. With the volume of the simulation boxed used, 10 API equals 2 mM, which is higher than the measured solubility in HIF for any of the APIs used. The *n* = 50 value was chosen to stress test our method.

The 25 final frames of the simulations were analyzed by calculating the number of contacts between API–beads and colloid–beads, divided by contacts between API–beads and water–beads. The ratio of these contact numbers was plotted to compare with the API solubility values reported in the literature, in fed HIF, and in water. This was a protocol similar to the one in our previous studies in the fasted state to explore solubilization capacity.^27^ The contacts calculations are made with the assumption that the apparent solubility will be increased by solubilization and that a high ratio between API–colloid over API–water indicates a high solubilization. The ratios from the simulations are then compared to the solubility enhancement; the ratio of solubility in fasted-state HIF over the aqueous solubility.

### 2.6 Analysis, Statistics, and Tools

Colloids were analyzed with an in-house Python script, as described in Parrow et al.;^27^ molecules were assigned to a colloid if they were within 0.5 nm of another molecule. If obvious clustering of colloids occurred, such as micelles close to each other, manual corrections of cluster assignments were made. Shape factors were calculated as the ratio between the largest and the smallest moments of inertia, where a perfect sphere would have a value of 1; i.e., the less spherical, the larger the value would be. Maximum diameter (*D*_max_) was calculated as the greatest distance between two beads within a colloid, and aggregation number (*N*_agg_) as the number of its molecules.

For the different calculations, additional Python scripts were used that build upon the MDAnalysis package.^33^ Surface coverage was calculated using a modification of PYTIM,^34^ and contacts were calculated with the GROMACS command GMX distance. *P*-values were calculated with an ordinary one-way ANOVA with multiple comparisons when comparing colloidal compositions. When comparing API fed-state affinities, a Kruskal–Wallis test with Dunn’s multiple comparison was used.

## 3 Results and Discussion

### 3.1 Self-Assembled Colloids

**Figure 2:**
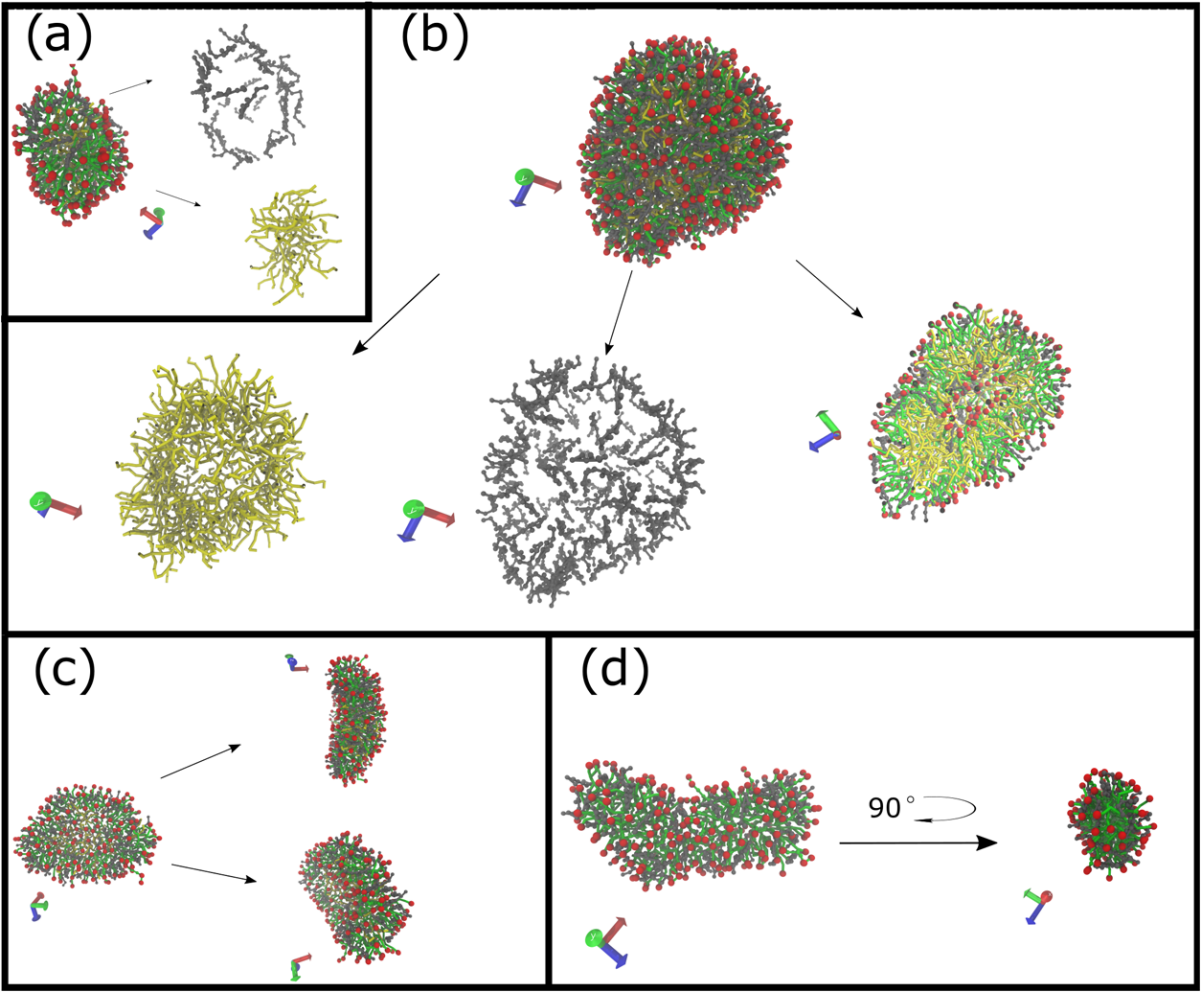
Types of colloids after 3 μs of simulation: (a) Prolate micelle with clear core–shell formation. (b) Vesicle (water beads not displayed) with water pocket in the core. (c) Oblate micelle formed in compositions of low amount of bile salts. (d) Elongated micelle, defined as micelles with shape factors over 2. Bile salts are colored gray, glycerides yellow, fatty acid head groups red, fatty acid tails green, phospholipids green.

The results of the three different simulation sets from the five HVs showed self-aggregation of four types: prolate (rugby ball-like), oblate (disk-like) and elongated (worm-like) micelles, and vesicles (Figure 2). Micelles (Figure 2a) with a prolate-ellipsoidal or spherical shape had a hollow bile salt shell at their surface, with the gaps covered by head groups of free fatty acids and phospholipids pointing outward. This is similar to what we have seen in fasted-state simulated micelles.^27^ The major difference was the inner core of glycerides, which possibly accounts for the larger size of fed-state micelles (4–19 nm) compared to the ones in the fasted state (2–7 nm). This is in the range of experimentally determined colloidal sizes in FeSSIF.^30,35^ In vesicles (Figure 2b), the free fatty acids were the main component that formed a bilayer, with glycerides and phospholipids inside, directing head groups outward. The inner core of the vesicles contained water beads. Bile salts did reside close to the free fatty acid head groups at the outer and inner layer surfaces facing the water beads. A more detailed description of the micelle and vesicle surface compositions is in the Supporting Information (Figure S1). The vesicles were larger than the micelles, both in terms of aggregation number (*N*_agg_) and diameter (*D*_max_), but with similar shape factors (Figure S2).

Two alternative micelle types to the prolate micelle were observed. The oblate micelles (Figure 2c) had a molecular disposition very similar to the prolate micelle type, except for fewer glycerides. We could also see elongated micelles (Figure 2d), which had a similar type of structure as prolate micelles, but with shape factor >2. Interestingly these structures were formed when no glycerides were present.

**Figure 3:**
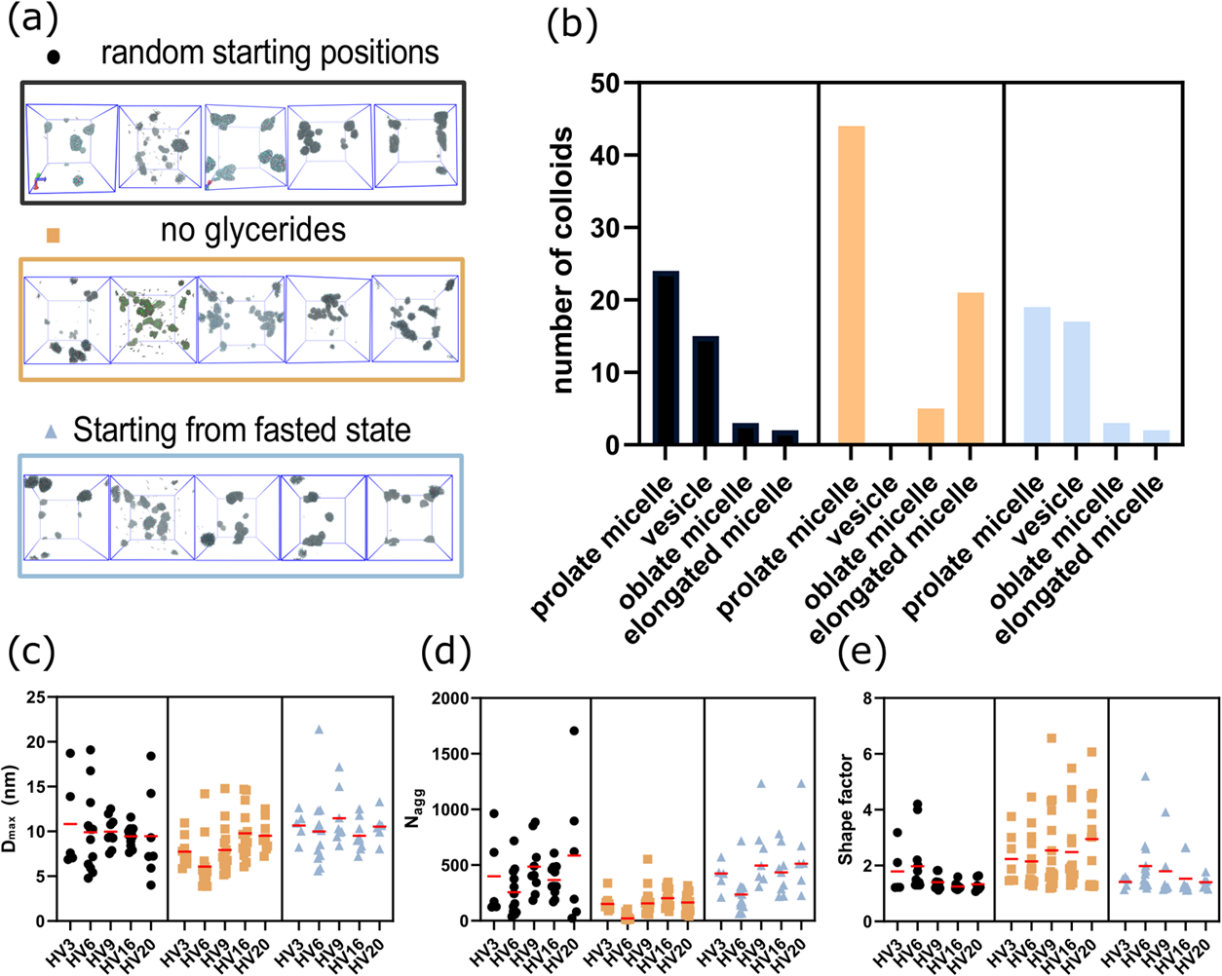
Results from three different starting sets of simulations. (a) Snapshots from the last frames of the three sets. (b) Number of colloids vs type. (c) Maximum diameter of colloids. (d) Aggregation number (*N*_agg_), i.e., number of molecules in each colloid. (e) Shape factor of colloid with a perfect sphere being = 1; the larger the value, the more elongated the colloid.

Intestinal fluid is known to have both high polydispersity, and inter- and intravariability of its small molecular composition.^36^ The polydispersity is qualitatively captured in the simulations when looking at size and shape of the assembled colloids. The final frames from the three simulation sets are shown in Figure 3a, and the number of different colloids per set in Figure 3b. In our simulations, the colloids had a size range of 4–19 nm, 15–1700 molecules (*N*_agg_), and shape factors between 1.2 and 7 (Figure 3c–e). Vesicles and prolate micelles were the most commonly found colloids in the systems and constitute the vast majority in two out of three simulation sets (Figure 3b). In systems without glycerides, no vesicles were formed. Instead the molecules self-assembled as elongated micelles (shape factor > 2). This clearly shows that the glyceride component was crucial for the vesicle formation in these simulations. Overall, the colloid types resembled those determined experimentally by cryo-TEM.^31,37^ However, the computational simulations deviate from the experimental colloids in that the box sizes possible to simulate for the time being may limit the size of colloids. Thus, the colloidal sizes are typically much larger in experimental characterizations. Apart from the limited box size, another possibility is that the smaller colloidal size in the simulations is a result of the force field or time scale used. We also noticed that colloids tended to cluster in some of the simulations, which could have impacted the shapes at the end of the simulations. It is well known that the Martini v2, which we used here, has a bias toward “sticky” beads.^38−40^ This bias is supposedly reduced in Martini v3,^41^ and hence, will be used in our future studies exploring the aggregation size and N_agg_.

A parameter that we aimed to evaluate was the impact of the starting configurations on simulation outcome (sets 1 and 3), and the impact of glyceride exclusion (set 2). Simulations with random starting positions and those starting from fasted state generally had similar results. The only differences in colloid characteristics were that slightly more vesicles formed in the systems starting from fasted state and that shape factors and colloid size differed a little comparing HVs intravariability across the three sets. This indicates that starting the simulations from the fasted state does perform as well as a randomized setup. The average *D*_max_ of the colloid at an individual level, for sets 1 and 3 were similar for all five HVs (Figure 3c–e). However, one clear difference emerged for HV6 compared to the other HVs; here there was a high concentration of monomeric bile salts and no vesicles were found (Figure S3). This was most likely due to the bile salt concentration leading to a higher ratio of bile salt to phospholipid, free fatty acid or glyceride ratio in HV6, compared to the other explored individuals (Table 1). The absence of vesicles in HV6 is in line with experimental characterizations by cryo-TEM, which had been performed on aspirated samples from the five HVs. In the cryo-TEM visualization of the fluids, HV6 had only micelles whereas vesicles were found in the other HVs,^31^ as was the case for our simulations. However, cryo-TEM detected micelles of 10–150 nm, whereas in our simulation, these were under 20 nm as a result of the limited box size possible to investigate.

Most of the *D*_max_ and *N*_agg_ values showed high polydispersity in all sets, as did the shape factors in simulation set 2. This makes sense since set 2 looked quite different from the other sets, due to the absence of vesicles and the presence instead of elongated micelles. The absence of glycerides reduced their hydrophobic cores enough that the micelles could not swell in all directions, leading to inhibition of more spherical colloids, seen in simulation sets 1 and 3. Bile salts, phospholipids, and free fatty acids could only continue assembling by elongating the micelles. This is reflected by the lower *D*_max_, *N*_agg_, and the higher shape factors.

**Figure 4:**
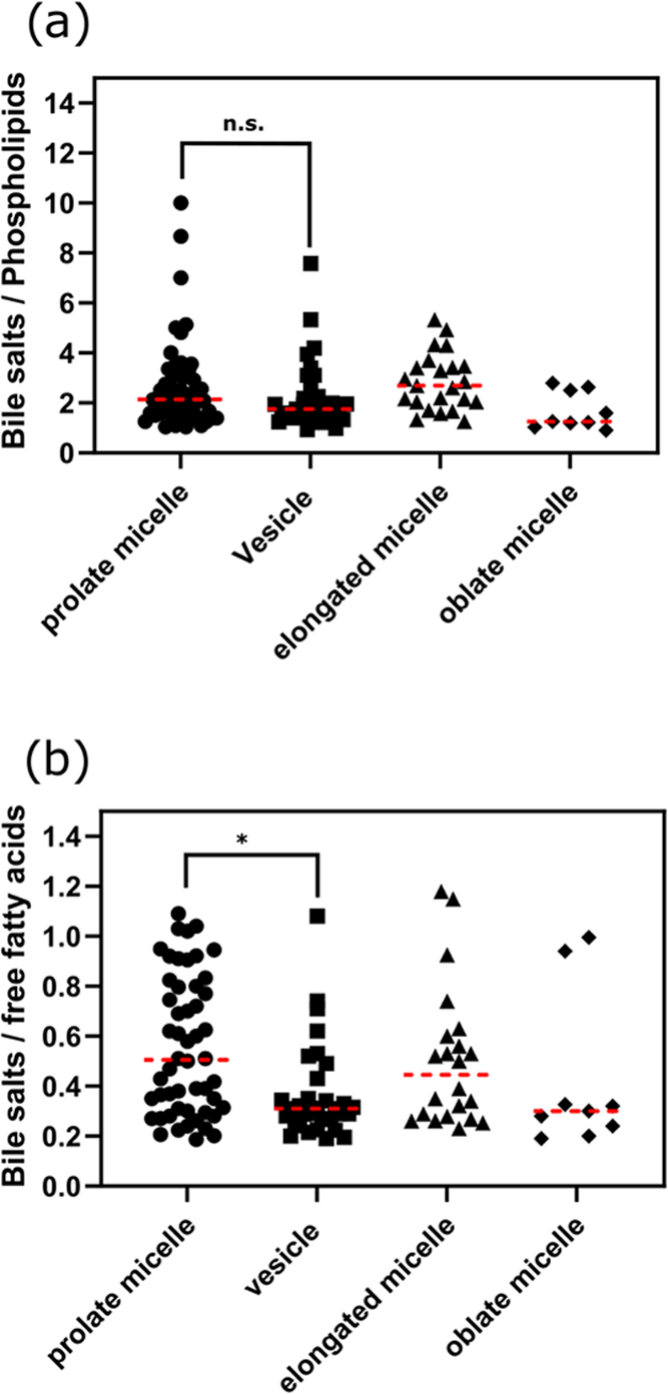
(a) Ratio of bile salts to phospholipids and type of colloids. No significance (n.s.) between prolate micelles and vesicles. (b) Ratio of bile salts to free fatty acids and type of colloid. The difference between prolate micelles and vesicles is significant (*p* = 0.0084, one-way ANOVA).

Simulations of intestinal fluid often take into account the ratio of bile salt to phospholipid to set up systems that correctly reflect the concentrations that would result in the formation of colloidal structures. By analyzing the composition of colloids from these simulations, we could see that the bile salt-to-phospholipid ratio did not always dictate the outcome. In our simulations, the prolate micelles and vesicles had similar ratios of bile salt to phospholipid, but a significant difference (*p* = 0.0084) in the ratio of bile salt to free fatty acids (Figure 4). The simulation results thus advocate that—even when the phospholipid-to-bile salt ratios are similar—the type of colloid formed could very well be dictated by the number of free fatty acids. The concentration of free fatty acids is not firmly linked to the phospholipid concentrations, according to data from a previous study; see examples in Table 1.

### 3.2 Absorption and Digestion

To mimic the impact of absorption and digestion we specifically simulated the aspirated in vivo composition from HV3 at three time points: 10, 40, and 90 min after ingestion of a liquid meal (Ensure Plus); see Table S1. HV3 was chosen since it contained all colloidal types and did not have an extreme bile salt-to-lipid ratio. Colloidal size and shape tended to decline over time for the three simulated time points (Figure S4). To further understand the effect of absorption on the colloids, we performed simulations in which free fatty acids were removed step by step from an isolated prolate micelle, a vesicle, an elongated and an oblate micelle. For the prolate micelle and vesicle, there were no drastic changes after removal of free fatty acids (Figure S5). The oblate micelle, however, transformed to a prolate shape, and similar structural changes were observed for the elongated micelle. The elongated micelle shrunk significantly in its longest direction, also leaving a few monomeric bile salts apart from the colloid itself at the end of the simulation (Figure S6). This rearrangement implies that the elongated and oblate micelles are greatly dependent on the free fatty acid concentration.

**Figure 5:**
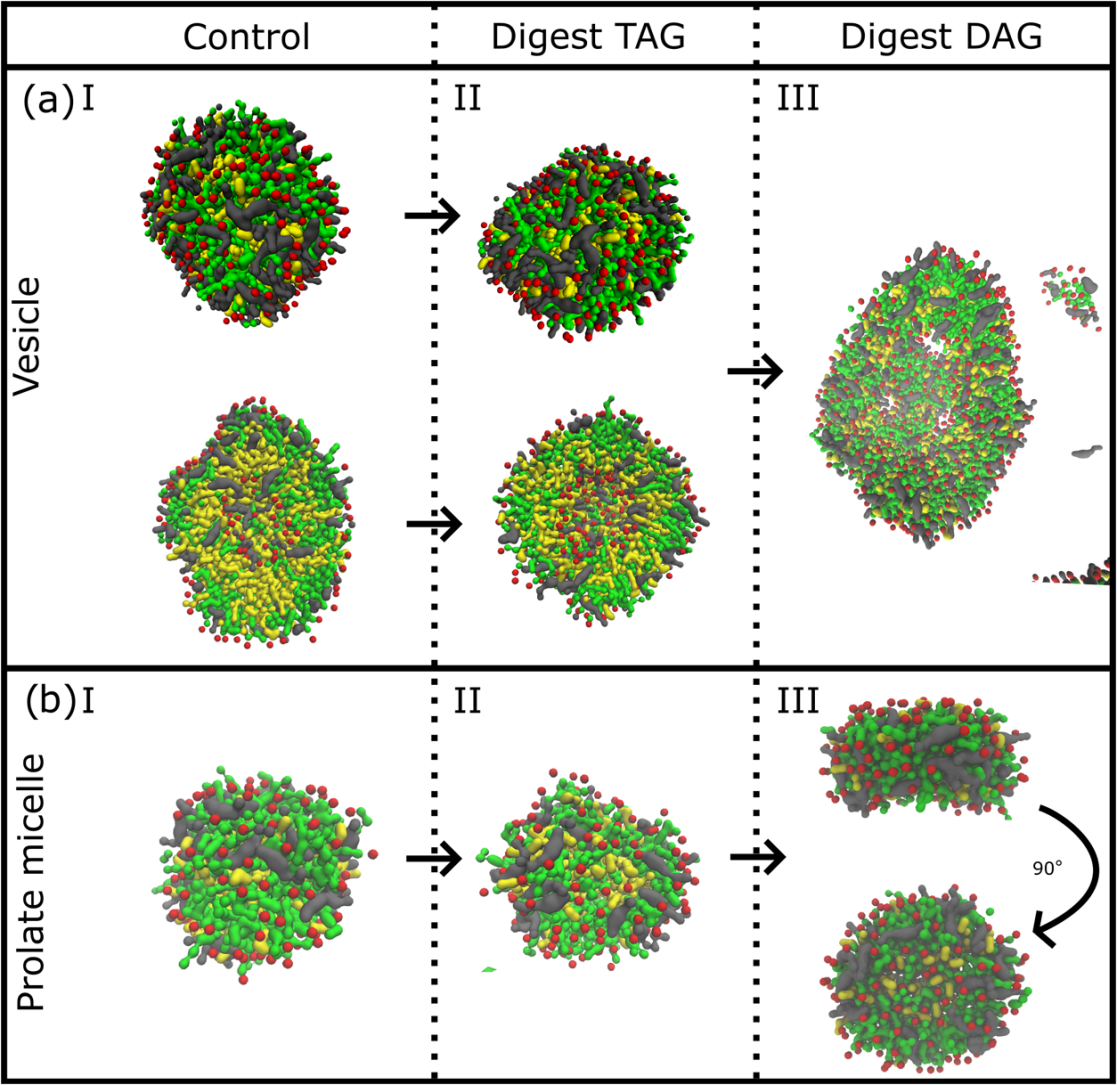
Simulation of a vesicle (a) and a prolate micelle (b), without (I) and with (II, III) digestion of glycerides. Bile salts are colored gray; glycerides, yellow; fatty acid head groups, red; and tails and phospholipids green. In (a) I and II, the vesicle is further displayed as cut in half to visualize the vesicular structure. Water beads are not shown.

In a similar manner to recognize digestion, glycerides were replaced by free fatty acids as described in the Materials and Methodssection. This was only performed on glyceride-containing colloids (the most common colloidal type); one prolate micelle and one vesicle were chosen. The vesicle and the prolate micelle shrunk, slightly if at all, while maintaining their shapes after digestion of TAG to one DAG and one free fatty acid. This was not the case when further digestion of DAG was simulated. After digestion of DAG to two free fatty acids, the vesicle was no longer stable; it disintegrated (as seen in Figure 5a) and lost its spherical and vesicular structures with the core of water beads. Digestion of DAG in the prolate micelle re-arranged the colloid into an oblate shape (Figure 5b). These colloidal readjustments due to composition changes again point out the importance of glycerides for vesicle assembly and the effect of free fatty acid concentration on micelle shape in these simulations. It is possible that a more biorelevant digestion method, one DAG to one MAG and one free fatty acid, would give a different outcome in the simulations, in particular for the digestion of the vesicle that perhaps could be stabilized better by MAGs and hinder the disintegration.

### 3.3 API–Colloid Interactions

**Figure 6:**
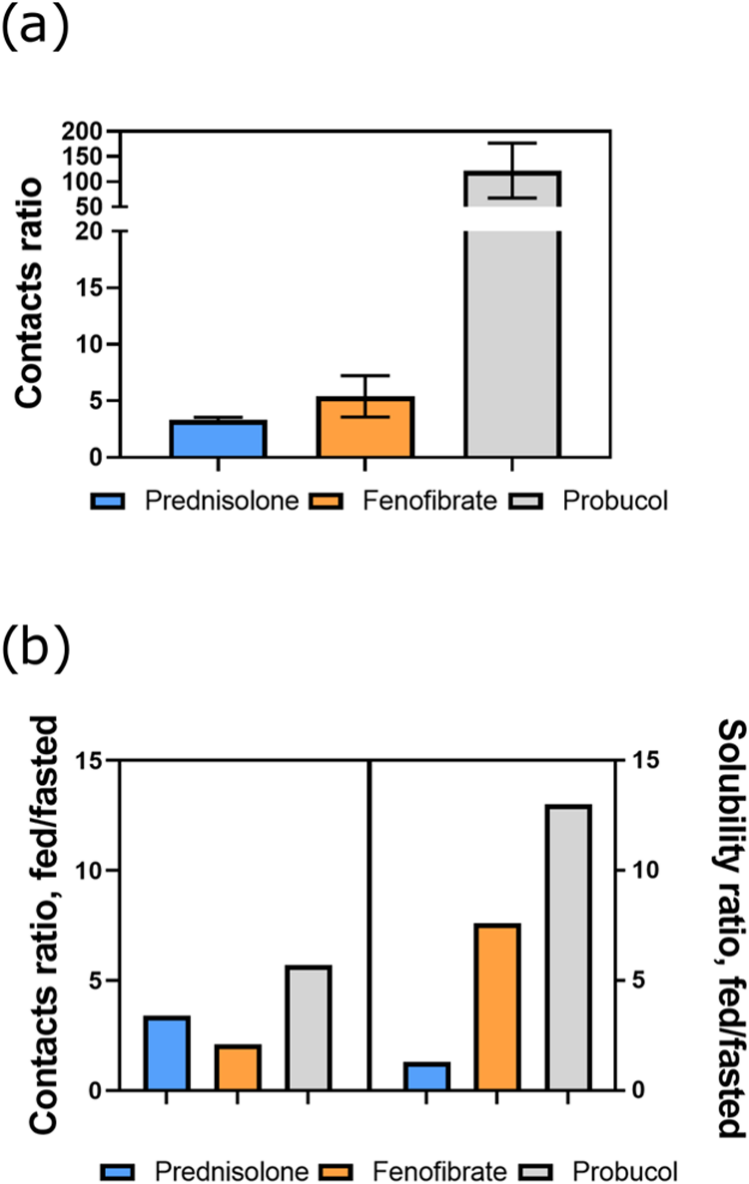
(a) Colloidal affinity of API, calculated using API–colloid contacts during the end of the simulations. (b) Comparison of colloidal affinity ratio between fasted and fed simulations with the literature values of fasted and fed solubility ratio.

We next attempted to assess API solubility from the simulations using the number of contacts between self-assembled colloids and APIs. One prolate micelle and one vesicle were isolated in new simulation boxes containing prednisolone, fenofibrate, or probucol. To link the interactions to solubility, we calculated the contacts between drug–colloid and drug–water. Our hypothesis was that the ranking of these contact ratios would be the same as the apparent drug solubility enhancement.^27^ The contact ratios calculated after 3 μs of simulation, of systems with 2–10 mM, are displayed in Figure 6a. As seen from the contacts analysis, the API rank order for colloidal affinity was (in decreasing order) probucol, fenofibrate, and prednisolone. A significant difference was only observed between probucol and the other two APIs (*p* < 0.002). The rank order of contact ratios was the same for both the micelle and the vesicle (Figure S7). Both micelles and vesicles are present after intake of food, and hence, the average was chosen to represent the fed state; see Figure 6a. The rank order that we obtained this way was in agreement with literature data of API solubilities (Table S2) in water and in HIF.^11,42,43^

However, we also wanted to compare the fed-state results to our earlier fasted-state results. The fasted-fed contact ratio from our simulations and the solubility ratio of APIs in fasted and fed state from the literature, are presented in Figure 6b. The literature solubility ratio for the three APIs had the same rank order as in our fed-state simulations. All APIs had higher contacts ratios in the fed-state simulations than those in the fasted state. However, the fed-fasted contact ratios from our simulations did not match with the solubility ratios. In our simulations, prednisolone had a slightly higher fed-to-fasted ratio than fenofibrate. This discrepancy indicates that the method used here is not precise enough to compare APIs in fasted and fed-state HIFs. It could be that these complex, polydisperse fluids require more than two simulated colloids to represent the solubility capacity accurately.

**Figure 7:**
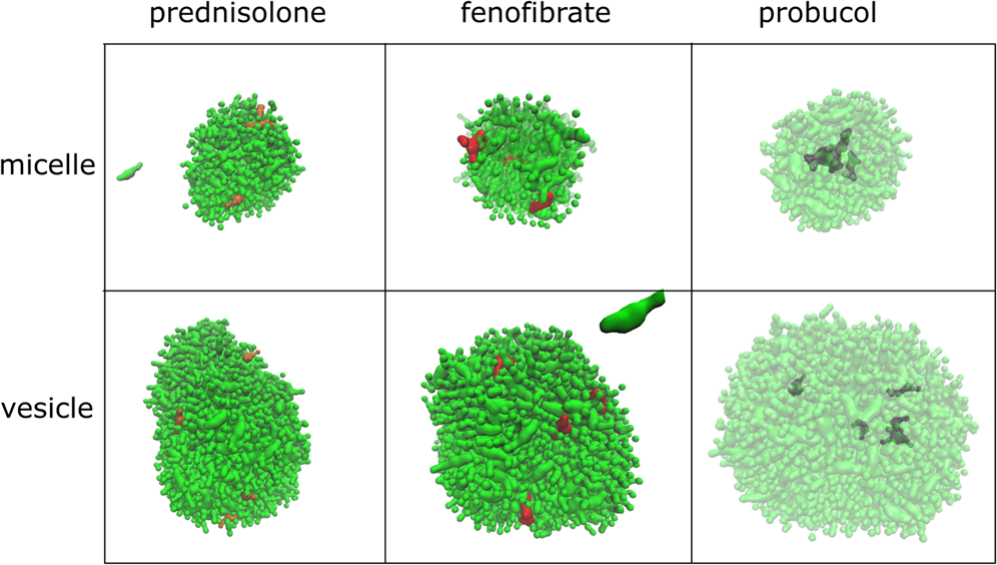
Snapshot from the end of the simulations of micelles and vesicles with API. Micelle and vesicle molecules are colored green; prednisolone, orange; fenofibrate, red; and probucol, gray. For simulations with probucol, the colloid molecules (green) are made transparent since probucol is positioned inside the colloids.

**Figure 8:**
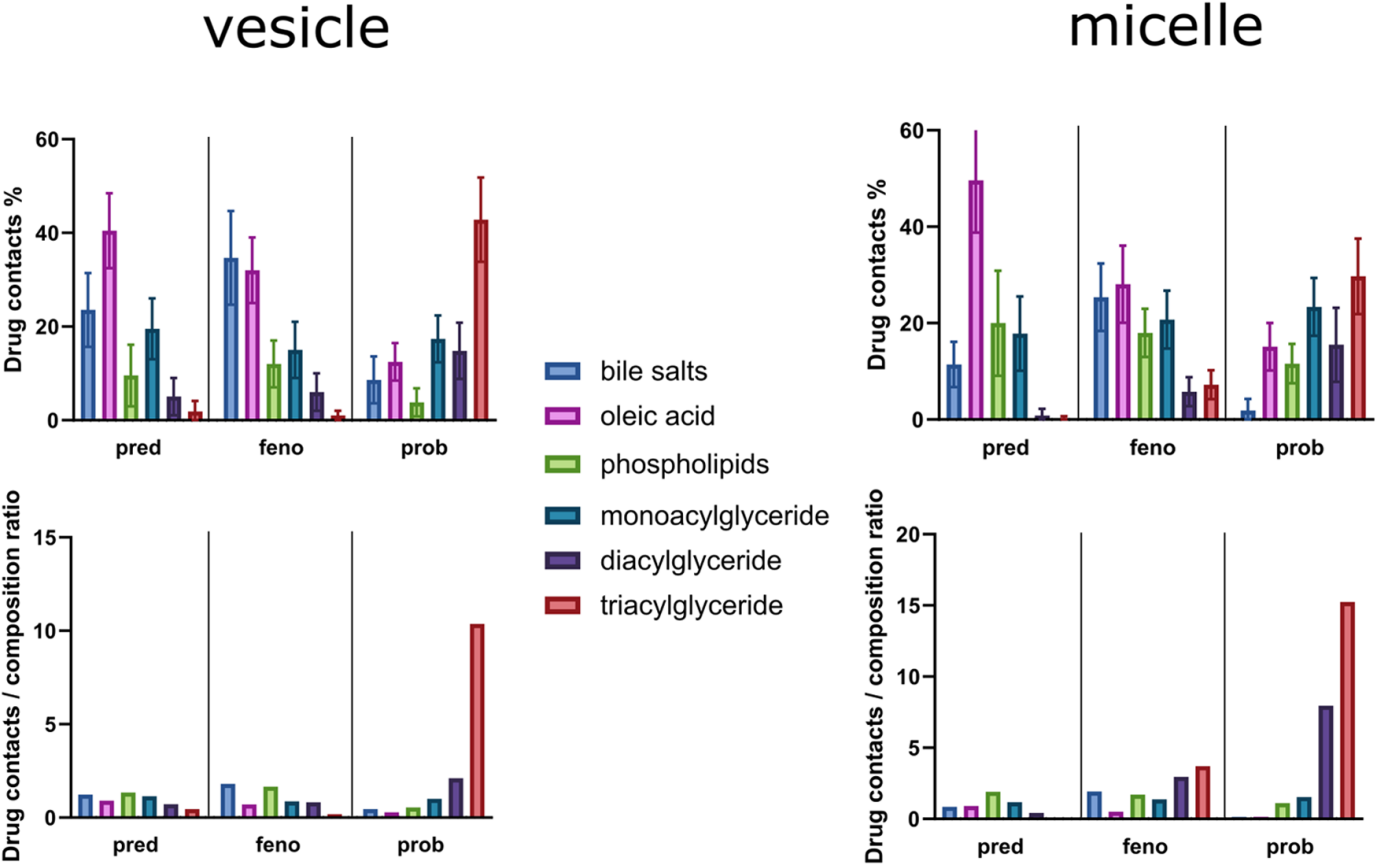
API contacts for specific molecules at the end of simulations. The bottom graphs are normalized based on the composition ratio in the colloid.

Using the simulations with APIs, we also looked closer at the specific interactions that occurred. The final frames of the API–colloid simulations are presented in Figure 7 (and from all simulations in Figure S8). Here, we saw that both prednisolone and fenofibrate are positioned at the surface (at least partially), while probucol resides in the core of the micelle, or in the glyceride layer of the vesicle. For the micelle, this was expected since a similar pattern has been observed in our simulated fasted-state micelles. To further understand how the APIs interact with the colloids, we looked at the specific contacts for the APIs (Figure 8a). In the micelle, prednisolone, which has the lowest log *D*, formed the most contacts with free fatty acids. Prednisolone was followed by fenofibrate; it had many contacts with free fatty acids, but even more with bile salts. Probucol, on the other hand, had half the number of contacts with free fatty acids, almost none with bile salts, but a relatively high number with TAGs. The strength of interactions with TAGs is further displayed by normalizing the number of contacts with the composition ratio for each specific HIF component (Figure 8b). The trend described for the micelle also can be seen for the vesicle, but with some differences. For instance, bile salt interactions with probucol were much stronger in the vesicle. This could be due to the inner water surface that the bile salts also cover, thereby enabling these interactions. Probucol also had fewer interactions with phospholipids in the vesicles; however, this could be simply because the vesicle has a lower phospholipid content compared to the micelle. The contacts were calculated from simulations with *n* = 5 API molecules. When *n* = 50 molecules, the APIs shifted toward more contacts with the free fatty acids (Figure S9). Micelles had slightly higher regions of bile salts covering the water surface than vesicles (Figures S1 and S10). An increase of APIs from 5 to 50 forced more of the API molecules to the surface of the colloids, especially in the micelles.

The results in Figures 7 and 8 can be compared to a recent study by Schlauersbach et al.,^44^ who introduced a solubility prediction tool based on descriptors and H-NMR shifts representative of interactions with either bile components (taurocholate and phospholipids) or lipids (monoglycerides). In this study, prednisolone was classified as bile interacting and nonlipid interacting, whereas fenofibrate was classified as both bile and lipid interacting. This is in agreement with our simulations when looking at the positioning and molecule interactions of these two compounds in the micelle. Probucol was not found in their dataset; however, the closest structural compounds to probucol used (ceritinib, cinnarizine) were also found to be both bile and lipid interacting. This type of prediction modeling could potentially make use of results from MD simulations as additional descriptor inputs.

## 4 Conclusions

We used CG-MD (with Martini force field v2) to simulate the intestinal fluids, using concentrations determined in aspirated samples from five different HVs after ingestion of a liquid meal. Simulations showed four major types of colloids: prolate, oblate, and elongated micelles, and vesicles with water-filled core. Colloids were from 4 to 20 nm, which is larger than micelles achieved in fasted-state simulations. Colloidal type depended on the molecular composition of the systems but not on the initial distribution of the molecules within the systems. We further attempted simulations mimicking the impact of digestion and absorption on the assembled colloids. Here, prolate micelles were generally less sensitive to free fatty acid concentration than the other micelle types. The prolate micelle was also less sensitive to digestion of glycerides than the vesicle. To see how assembled colloids would solubilize APIs with low water solubility, we added prednisolone, fenofibrate, or probucol to the simulated vesicles and micelles. The contact ratios of API–colloid to API–water were calculated as a solubility proxy and were in line with experimental solubility enhancement in the literature. Hence, this work shows that the coarse-grained MD simulation methodology is a promising tool for investigation of the intestinal fluids, in terms of colloidal attributes and drug solubility. Dissimilarities were mainly seen when comparing the fed-/fasted-state ratios between prednisolone and fenofibrate, where the literature data clearly states that fenofibrate has a higher ratio than prednisolone. If this is due to an overestimation of solubilization in the fasted-state simulations of fenofibrate or an underestimation in fed-state simulations, or inverse for prednisolone, is difficult to determine from the methods used. It is not certain if it is the simulated colloids or the APIs that has to be improved; this could be further investigated with other API molecules with validated topologies and solubility data. Also, for a better representation of fed/fasted solubility ratios, an extrapolation to initial simulated system size (or number of colloids) could be performed.

## Supporting Information Available

Analysis of bead distribution at the water surface of a prolate micelle and vesicle; colloidal characteristics from simulations of HIF-samples; simulations of digestion and absorption not included in the main manuscript, with concentrations used; snapshots of last frames from all simulations with API molecules; graphs of contacts ratio of micelle and vesicle; distribution of molecules at the surface of the micelle and vesicle used for API simulation; and solubility and log *D* values for the APIs used (PDF)
