# Supplementary material for: Molecular Dynamics Simulations of Self-Assembling Colloids in Fed-State Human Intestinal Fluids and Their Solubilization of Lipophilic Drugs
Source: Mol Pharm. 2022 Nov 9;20(1):451–60. doi: 10.1021/acs.molpharmaceut.2c00710 (PMC9811461; doi:10.1021/acs.molpharmaceut.2c00710)
Supplement: Supplementary file 1 — mp2c00710_si_001.pdf [file mp2c00710_si_001.pdf]

Molecular dynamics simulations of self-assembling colloids in fed state human intestinal fluids and their solubilization of lipophilic drugs

Albin Parrow[1], Per Larsson[1,2], Patrick Augustijns[3] and Christel A. S. Bergström[1,2]

1Department of Pharmacy, Uppsala University, Uppsala Biomedical Center P.O. Box 580, SE-751 23 Uppsala, Sweden

2The Swedish Drug Delivery Center, Department of Pharmacy, Uppsala University, Biomedical Center P.O. Box 580, SE-751 23 Uppsala, Sweden

3 Department of Pharmaceutical and Pharmacological Sciences, KU Leuven, O&N II Gasthuisberg, Herestraat 49, Box 921, 3000 Leuven, Belgium

Supporting information

Table of content

1. Colloidal beads at water surface
2. Colloidal characteristics
3. Impact on colloidal structures from digestion and absorption
4. API-colloid contacts analysis and molecules at the surface of colloids

5. References

1. Colloidal beads at water surface

To get an understanding of the micelle and vesicle conformation we calculated the number of beads at the water surface of the colloids. This was done with a python script build on PYTIM. The average percentages of surface presence during the last 125 ns are displayed in figure S1.

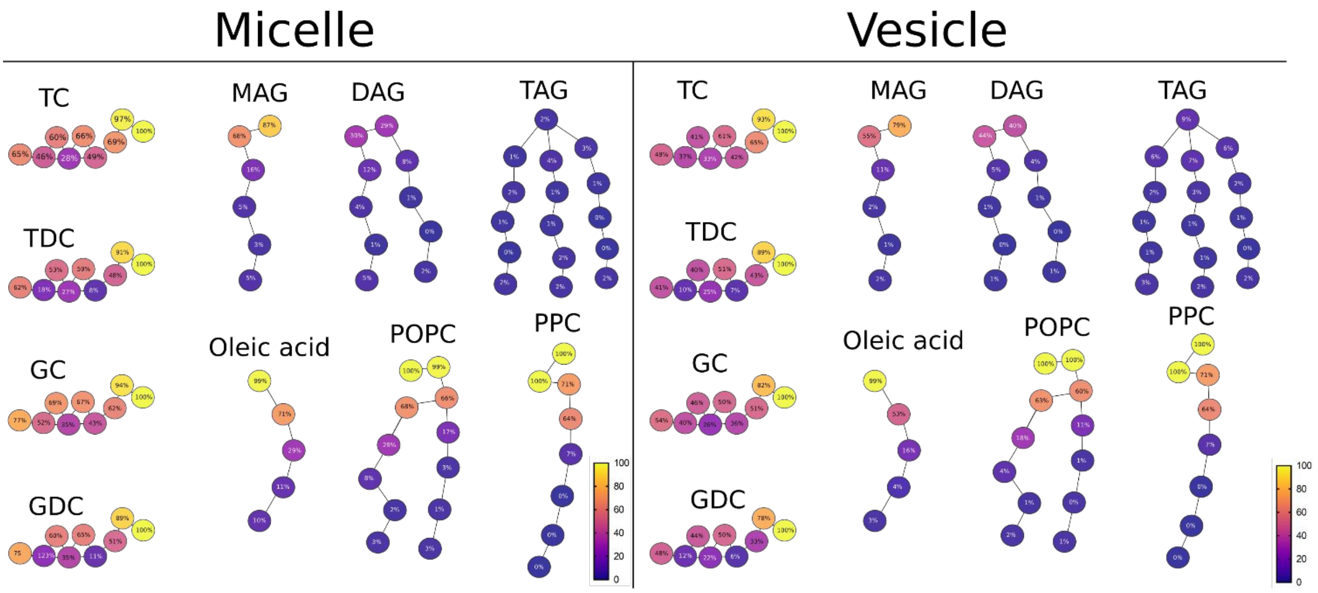

Figure S1. Description of beads at the water surface in the colloids during the last 125 ns. Yellow means that 100% of the specific bead are at the water surface, and blue means 0%.

2. Colloidal characteristics

Maximum diameter (D_{max}), shape factor and aggregation number (N_{agg}) were calculated as described in the method section. Values for micelles and vesicles, from all simulation sets, can be seen in figure S2. The number of different colloid types, for each HV, can be seen in figure S3.

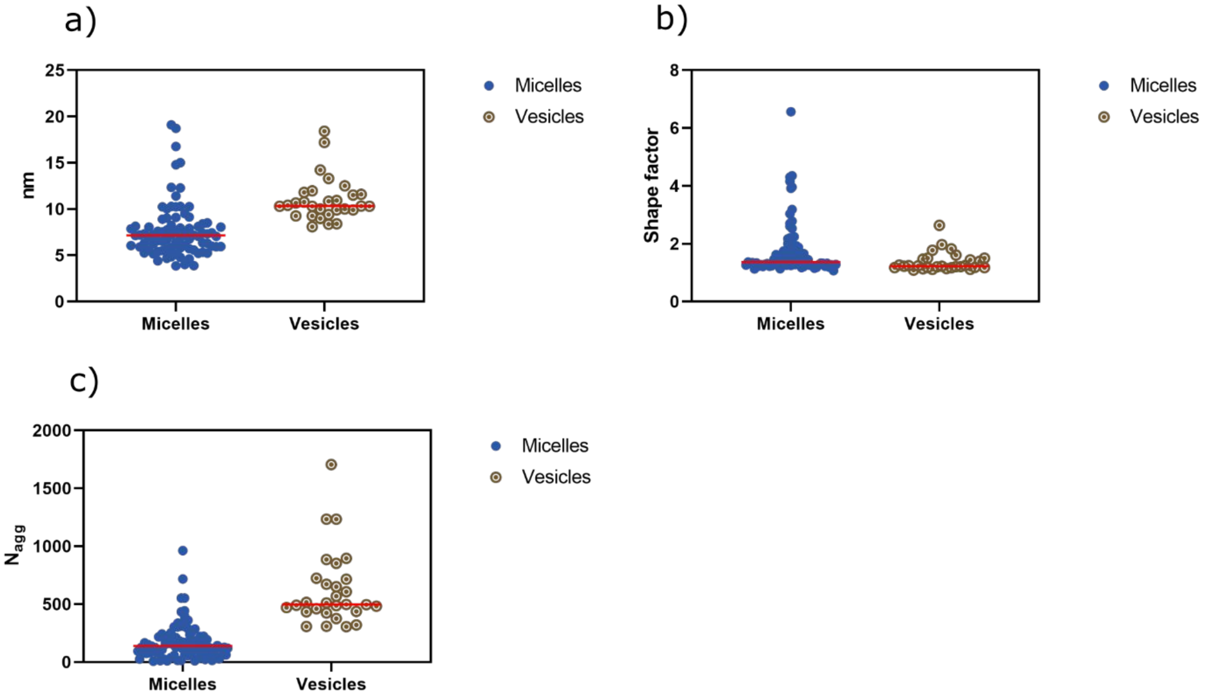

Figure S2. Colloidal characteristics of vesicles and micelles in terms of a) D_{max}, b) shape factor, c) N_{agg}. The median is marked with a red line.

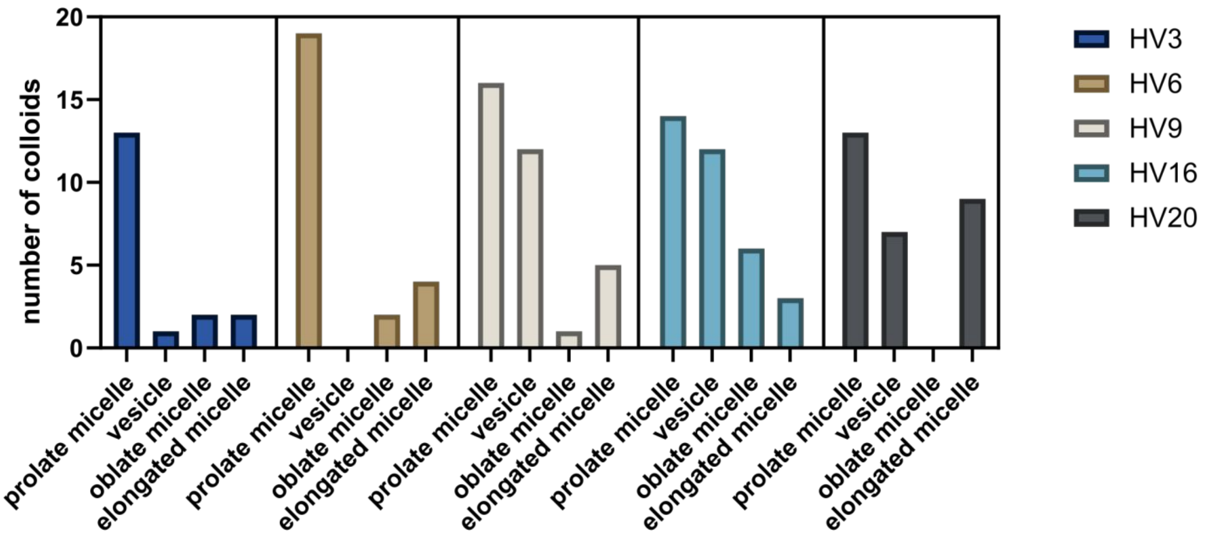

Figure S3. Number of colloids for each HV, from all simulation sets.

3. Impact on colloidal structures from digestion and absorption

Three different concentrations of aspirated samples from HV3, corresponding to 10, 40 and 90 minutes after ingestion of nutrient supplement, was simulated. Colloidal attributes can be seen in figure S4, and concentrations used in table S1. To mimic impact of absorption on specific colloids, simulations of single colloids were performed with removal of free fatty acids (absorption), according to the method section. In figure S5 a prolate micelle and vesicle are displayed before and after absorption. In figure S6 an oblate and an elongated micelle are displayed before and after absorption.

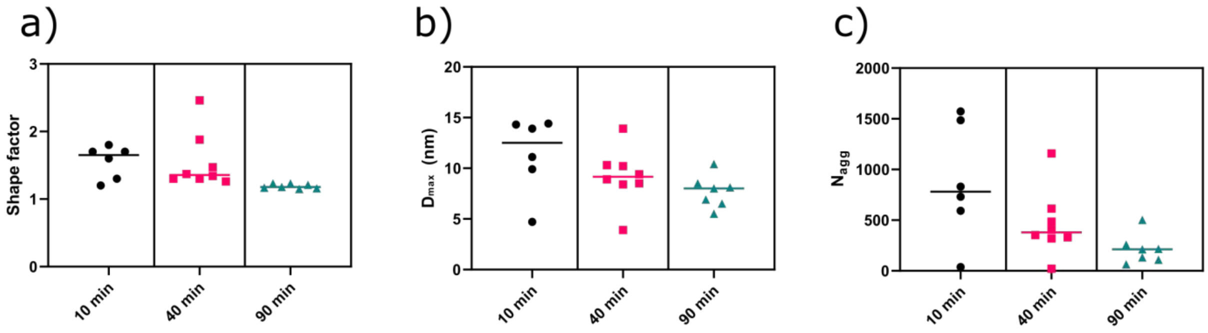

Figure S4. Colloidal attributes for HV3, simulated with reported concentrations at three different time points after ingestion of nutrient supplement.

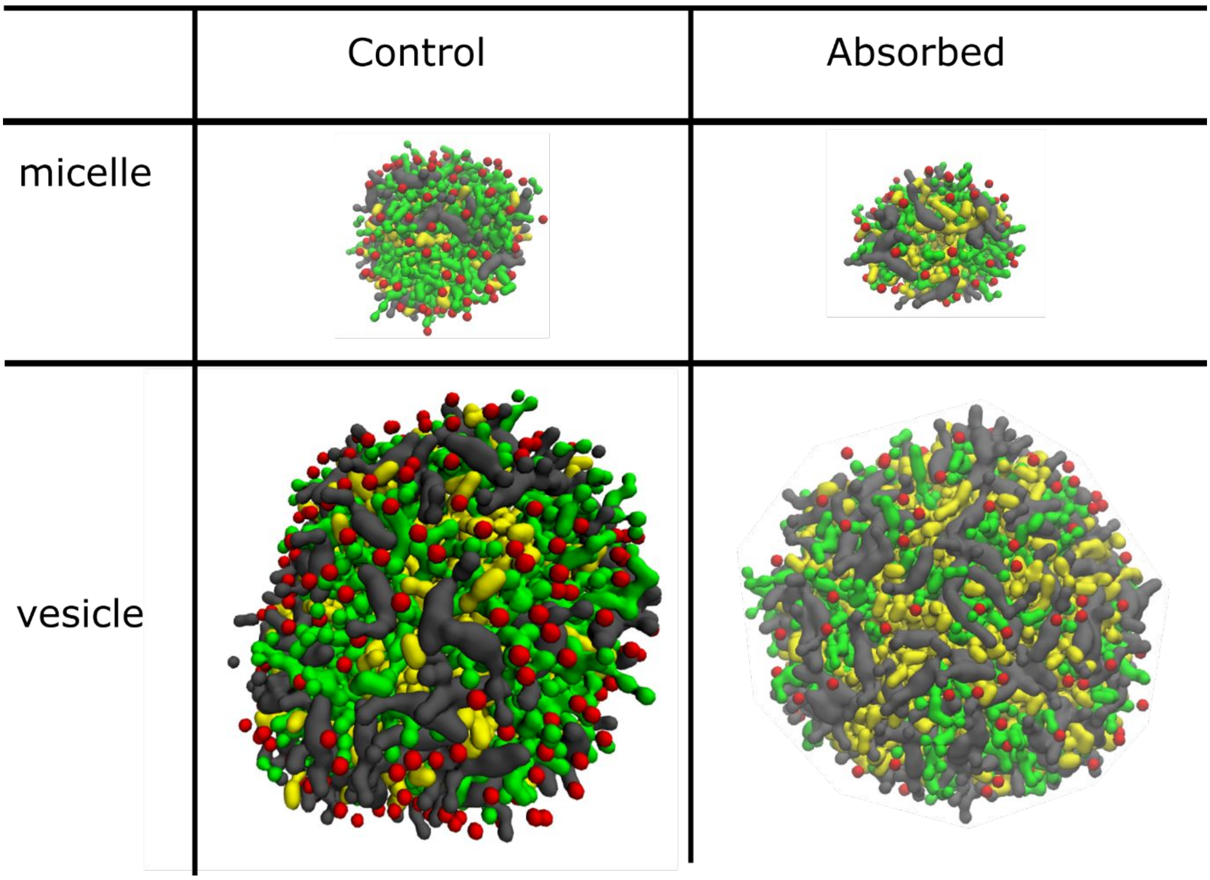

Figure S5. Vesicle and prolate micelle pre and post simulated absorption of free fatty acids. Bile salts are colored gray, glycerides yellow, fatty acid head groups red and tails green, phospholipids green.

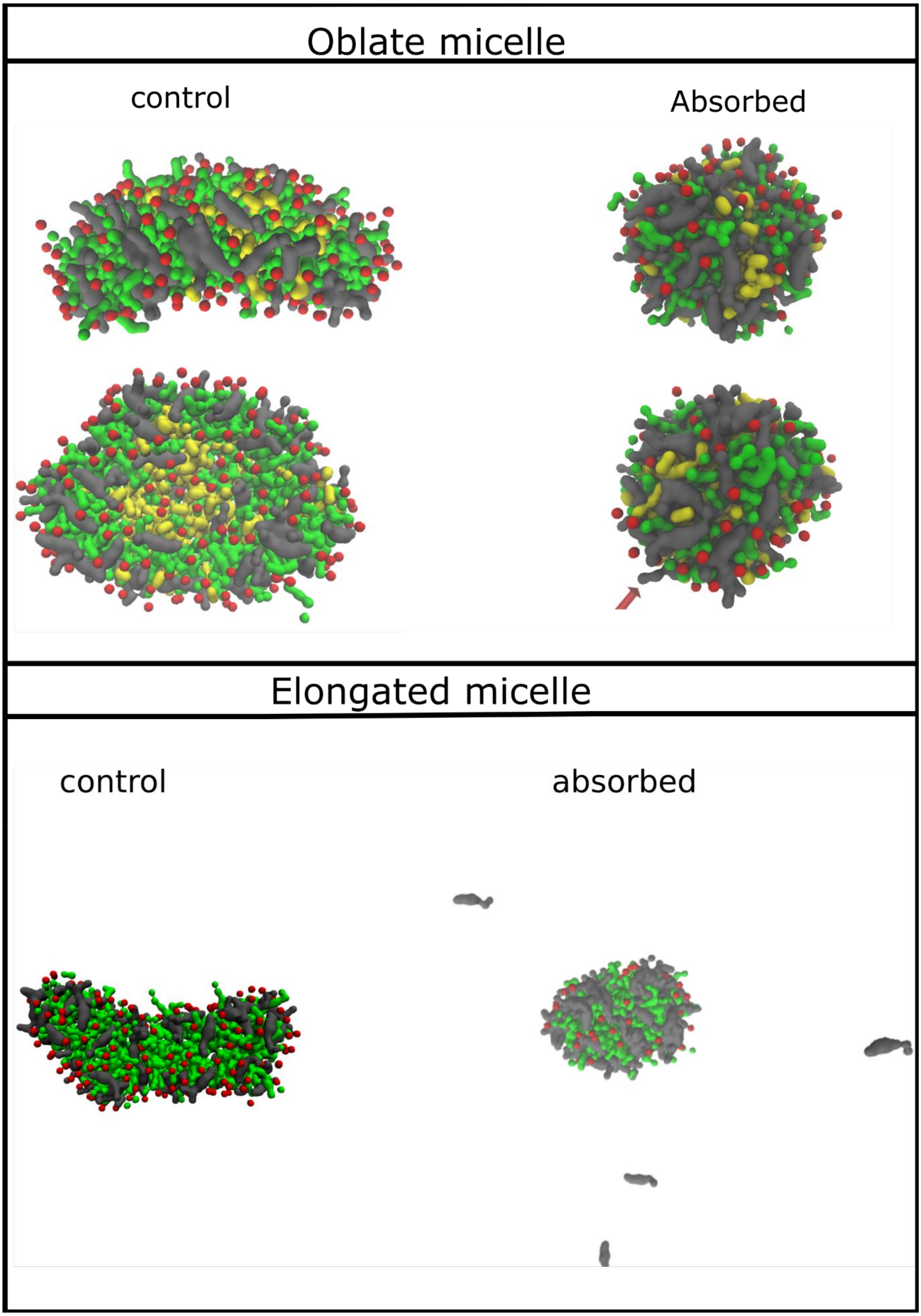

Figure S6. Oblate and elongated micelle pre and post simulated absorption of free fatty acids. Bile salts are colored gray, glycerides yellow, fatty acid head groups red and tails green, phospholipids green.

S1. Concentrations at time points

hv3 time points	Bile salts (mM)	PPL(mM)	Cholestrol (mM)	FFA (mM)	MAG (mM)	Dag	TAG
t10	20.7	2.92	1.71	21.60	14.17	2.40	0.36
t40	4.16	1.88	0.54	13.31	4.94	2.04	0.39
t90	10.07	3.51	0.0013	47.65	26.46	5.19	2.95

4. **API-colloid contacts analysis and surface molecules in colloids**

Contacts between API and colloids with GMX distance during the last 125 ns. The ratio of contacts between API-colloid and API-water was calculated for both a vesicle and a micelle, the average values from three concentrations of APIs (n = 2, 5, or 10) are seen in figure S7. Last frame of simulations with APIs are displayed in figure S8. Specific colloidal molecules contacts with APIs, from simulations with 50 APIs, can be seen in figure S9. The percentage of molecules at the surface during simulations with 5 and 50 API molecules are seen in figure S10.

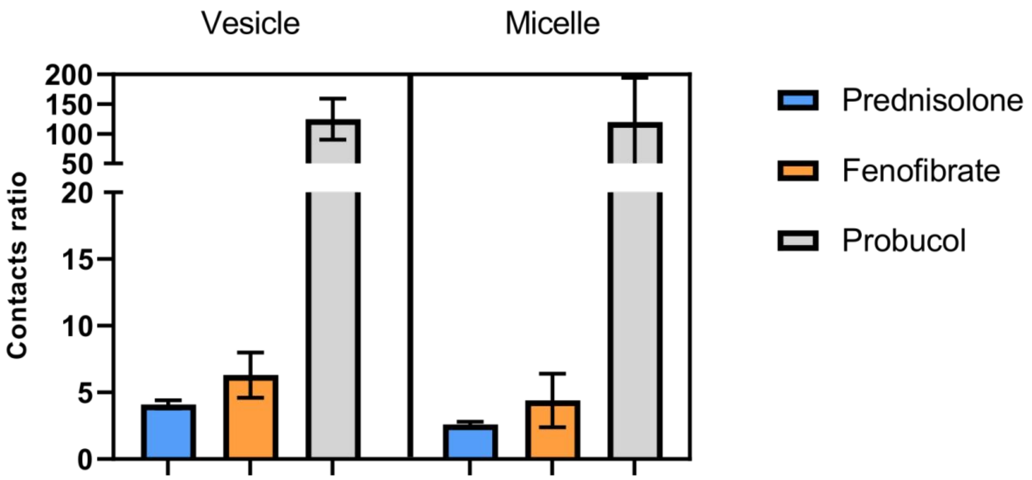

Figure S7. Contacts ratio (API-colloid to API-water) from simulations of vesicle and micelles with APIs.

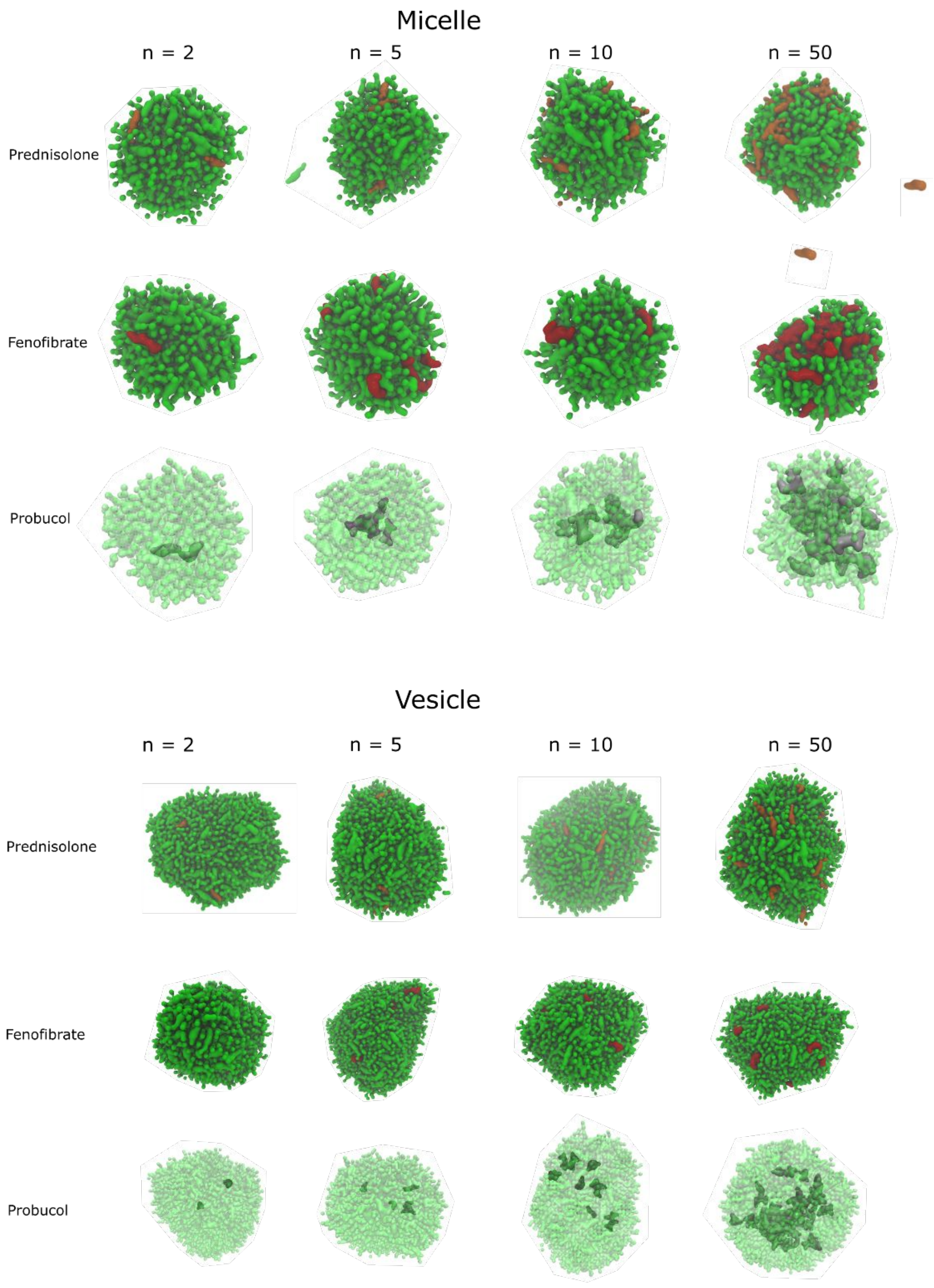

Figure S8. Snapshots from last frames of simulations of colloids with APIs at four different concentrations. Prednisolone is colored orange, fenofibrate red and probucol gray, colloid molecules are all colored green. The light color is indicating transparency since probucol is not seen on the surface of the colloids.

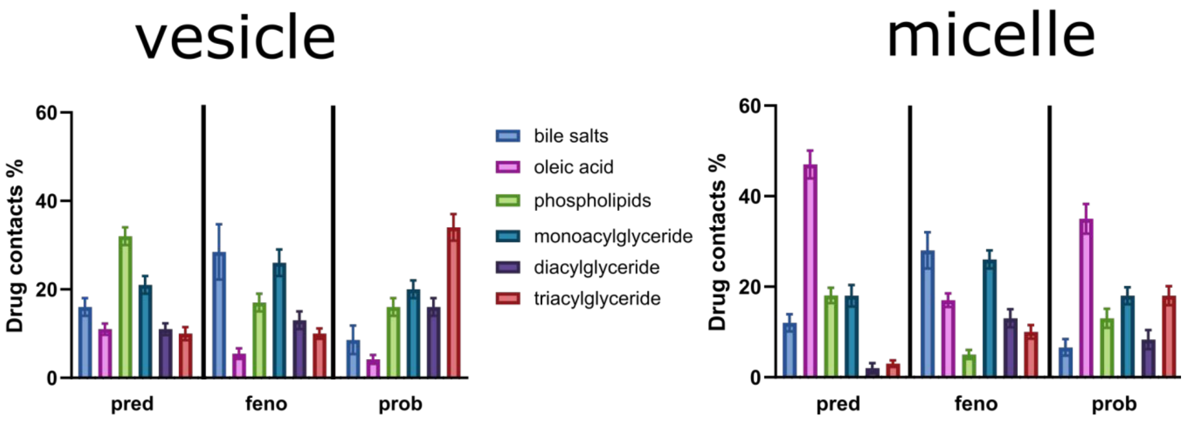

Figure S9. Contacts between API and colloid molecules, from simulations with 50 API molecules.

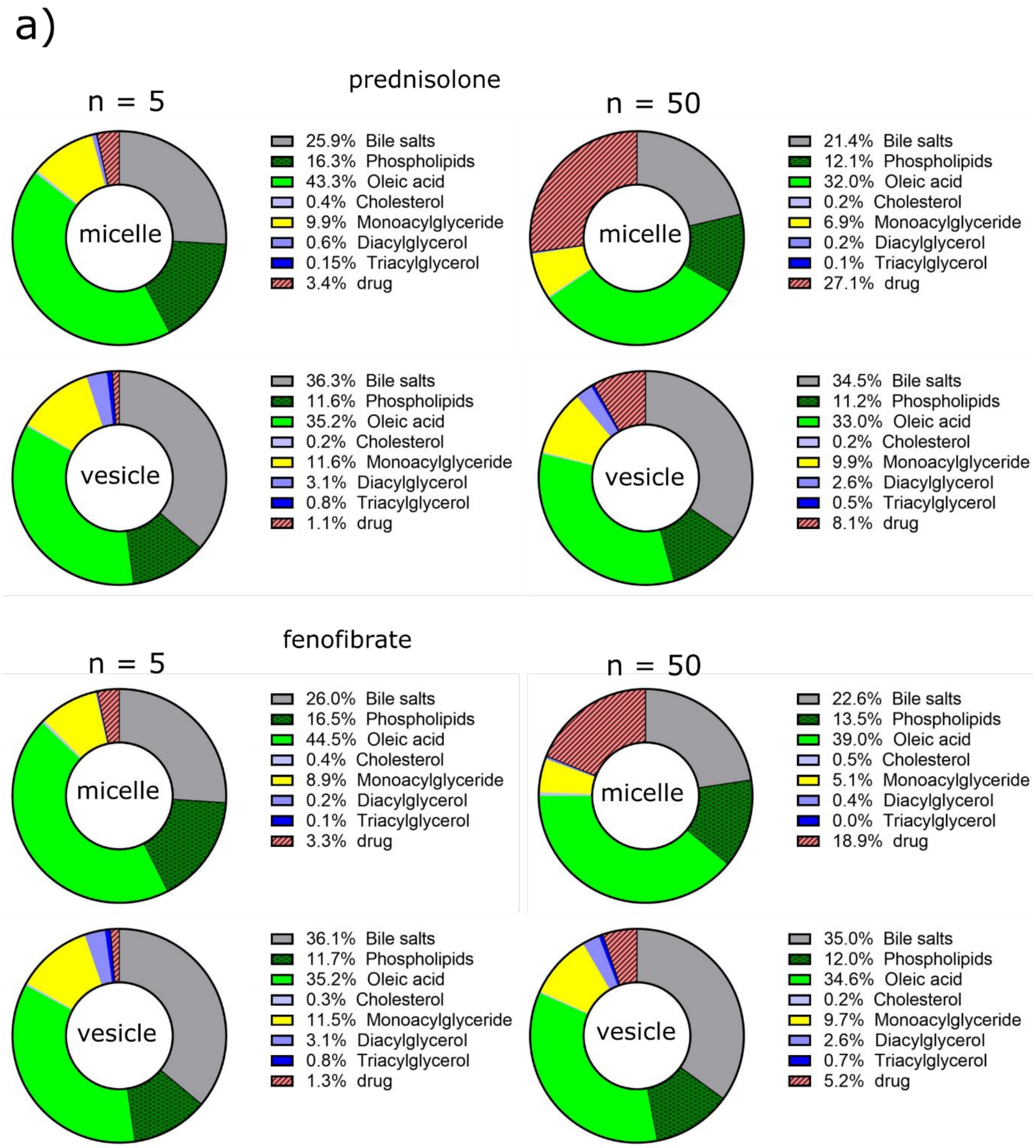
a)
prednisolone
n = 5
micelle
25.9% Bile salts
16.3% Phospholipids
43.3% Oleic acid
0.4% Cholesterol
9.9% Monoacylglyceride
0.6% Diacylglycerol
0.15% Triacylglycerol
3.4% drug
n = 50
micelle
21.4% Bile salts
12.1% Phospholipids
32.0% Oleic acid
0.2% Cholesterol
6.9% Monoacylglyceride
0.2% Diacylglycerol
0.1% Triacylglycerol
27.1% drug
vesicle
36.3% Bile salts
11.6% Phospholipids
35.2% Oleic acid
0.2% Cholesterol
11.6% Monoacylglyceride
3.1% Diacylglycerol
0.8% Triacylglycerol
1.1% drug
vesicle
34.5% Bile salts
11.2% Phospholipids
33.0% Oleic acid
0.2% Cholesterol
9.9% Monoacylglyceride
2.6% Diacylglycerol
0.5% Triacylglycerol
8.1% drug
fenofibrate
n = 5
micelle
26.0% Bile salts
16.5% Phospholipids
44.5% Oleic acid
0.4% Cholesterol
8.9% Monoacylglyceride
0.2% Diacylglycerol
0.1% Triacylglycerol
3.3% drug
n = 50
micelle
22.6% Bile salts
13.5% Phospholipids
39.0% Oleic acid
0.5% Cholesterol
5.1% Monoacylglyceride
0.4% Diacylglycerol
0.0% Triacylglycerol
18.9% drug
vesicle
36.1% Bile salts
11.7% Phospholipids
35.2% Oleic acid
0.3% Cholesterol
11.5% Monoacylglyceride
3.1% Diacylglycerol
0.8% Triacylglycerol
1.3% drug
vesicle
35.0% Bile salts
12.0% Phospholipids
34.6% Oleic acid
0.2% Cholesterol
9.7% Monoacylglyceride
2.6% Diacylglycerol
0.7% Triacylglycerol
5.2% drug

b)

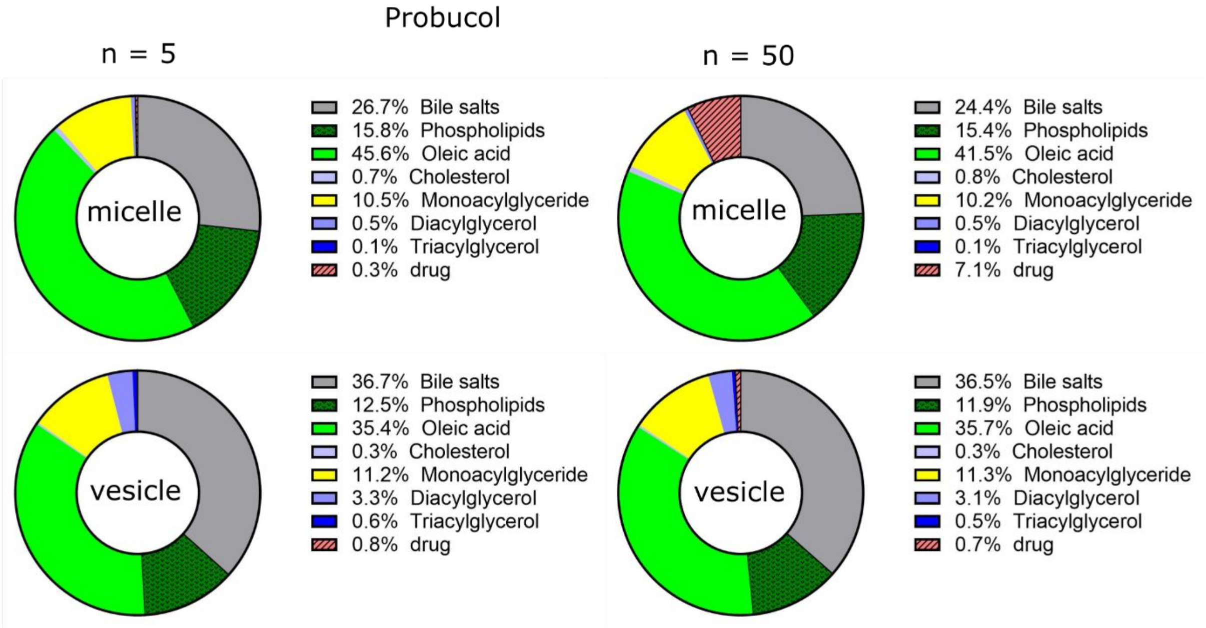

Figure S10. Molecules at the surface of micelles and vesicles during simulations with APIs.

Table S2. Calculated log D values for APIs used, and experimentally determined solubility of APIs in pH 6.5 buffer, fasted state HIF and Fed state HIF reported in literature [1–3].

	**Prednisolone**	**fenofibrate**	**Probucol**
**$logD_{pH6.5}$**	1.6	5.3	10
**$S_{pH6.5(\mu g/mL)}$**	375.9	0.3	0.006
**$S_{FaHIF(\mu g/mL)}$**	481.9	19.5	0.9
**$S_{FeHIF(\mu g/mL)}$**	507.8	147.6	32

5. References

(1) Clarysse, S.; Brouwers, J.; Tack, J.; Annaert, P.; Augustijns, P. Intestinal Drug Solubility Estimation Based on Simulated Intestinal Fluids: Comparison with Solubility in Human Intestinal Fluids. *Eur. J. Pharm. Sci.* **2011**, *43* (4), 260–269. https://doi.org/10.1016/j.ejps.2011.04.016.

(2) Söderlind, E.; Karlsson, E.; Carlsson, A.; Kong, R.; Lenz, A.; Lindborg, S.; Sheng, J. J. Simulating Fasted Human Intestinal Fluids: Understanding the Roles of Lecithin and Bile Acids. *Mol. Pharm.* **2010**, *7* (5), 1498–1507. https://doi.org/10.1021/mp100144v.

(3) Persson, E. M.; Gustafsson, A.-S.; Carlsson, A. S.; Nilsson, R. G.; Knutson, L.; Forsell, P.; Hanisch, G.; Lennernäs, H.; Abrahamsson, B. The Effects of Food on the Dissolution of Poorly Soluble Drugs in Human and in Model Small Intestinal Fluids. *Pharm. Res.* **2005**, *22* (12), 2141–2151. https://doi.org/10.1007/s11095-005-8192-x.
